# Glucose Management in the ICU: The Bi‐Centre GEM‐ICU Cohort Study

**DOI:** 10.1111/aas.70207

**Published:** 2026-02-19

**Authors:** Milda Grigonyte‐Daraskeviciene, Ruben Julius Eck, Morten Hylander Møller, Benjamin Skov Kaas‐Hansen, Morten Heiberg Bestle, Christian Lange Gantzel, Anders Granholm, Anders Perner

**Affiliations:** ^1^ Department of Intensive Care Copenhagen University Hospital—Rigshospitalet Copenhagen Denmark; ^2^ Department of Internal Medicine University Medical Center Groningen Groningen the Netherlands; ^3^ Department of Clinical Medicine University of Copenhagen Copenhagen Denmark; ^4^ Department of Anaesthesia and Intensive Care Copenhagen University Hospital—North Zealand Hillerød Denmark; ^5^ Section of Biostatistics, Department of Public Health University of Copenhagen Copenhagen Denmark

**Keywords:** critical illness, dysglycaemia, glucose, hypoglycaemia, insulin, intensive care unit

## Abstract

**Introduction:**

Dysglycaemia is common in intensive care unit (ICU) patients and has been associated with worse outcomes. Data on glucose management and insulin use from contemporary intensive care units (ICUs) are limited.

**Methods:**

We conducted a bi‐centre, retrospective cohort study of acutely admitted, adult patients at two Danish ICUs. Patients were included consecutively by backtracking admissions from December 1, 2023, until the target sample size (*n* = 300) was reached. Data were collected from electronic health records following a predefined protocol. The primary outcome was the occurrence of hypoglycaemia (< 4 mmol/L) during ICU stay. Secondary outcomes included severe hypoglycaemia (< 2.3 mmol/L), hyperglycaemia (> 10 mmol/L), time below blood glucose target range (< 6 mmol/L), time above target range (> 10 mmol/L), mortality at 30 days, days alive without life support at 30 days (DAWOLS) and days alive out of hospital at 30 days (DAOH). Process variables were also assessed.

**Results:**

We screened 311 ICU patients and included 300, of whom 285 had at least one blood glucose measurement. Hypoglycaemia was observed in 21 patients (7.4%; 95% CI 4.6–11.0) and severe hypoglycaemia in 3 patients (1.1%; 95% CI 0.2–3.0). Hyperglycaemia was observed in 186 patients (65.3%; 95% CI 59.4–70.8). Mortality at 30 days was 24.3% (95% CI 19.6–29.6). Median DAWOLS at 30 days was 27 days (95% CI 26–28; IQR 8–30) and median DAOH at 30 days was 9 days (95% CI 3–14; IQR 0–21).

**Conclusion:**

In this Danish bi‐centre cohort of acutely admitted ICU patients, hypoglycaemia was uncommon and severe hypoglycaemia was rare, whereas hyperglycaemia occurred in almost every second patient. Insulin was frequently administered, mostly intravenously, and glucose was monitored often. Patients receiving insulin spent nearly half of the observed time above the target range.

**Editorial Comment:**

In this retrospective analysis from 2 Danish centres, glucose management and insulin use in the ICU is presented. Rare hypoglycemia and more common hyperglycemia measurement events were counted, along with insulin administration, which was common.

## 1 Introduction

Hyperglycaemia is common in intensive care unit (ICU) patients due to acute metabolic stress and for some patients also due to pre‐existing diabetes [1, 2, 3, 4, 5]. Elevated blood glucose is usually managed with insulin, which poses a risk for iatrogenic hypoglycaemia [6, 7, 8]. However, both hyperglycaemia and hypoglycaemia are associated with increased mortality in ICU patients [9, 10, 11].

An early single‐centre randomised clinical trial in surgical ICU patients suggested that tight glycaemic control (4.4–6.1 mmol/L) with insulin improved survival [2]. However, these findings could not be replicated in a multicentre setting. The international “Normoglycemia in Intensive Care Evaluation–Survival Using Glucose Algorithm Regulation” (*NICE‐SUGAR*) trial showed increased 90‐day mortality amongst 6104 adult ICU patients where patients were allocated to tight versus conventional glycaemic control [3]. This trial set the basis for current clinical practice guidelines, which generally recommend initiating insulin therapy at blood glucose concentrations above 10 mmol/L [12, 13]. Although guidance on glucose targets for ICU patients exists [12, 13], no universally standardised or consensus‐based target has been established due to the absence of strong evidence.

Although contemporary ICU data on incidences of dysglycaemia are available [14, 15, 16, 17], Danish data describing glycaemic control and insulin use are needed for the planning of a randomised trial within this topic. We aimed to describe glucose management in two Danish ICUs, focusing on dysglycaemia, insulin use and management practices to inform the design of an upcoming randomised clinical trial. We hypothesised that the occurrence of hypoglycaemia was lower than that of hyperglycaemia in acutely admitted patients in the ICU. Moreover, we expected that glucose monitoring and insulin practices, particularly the route of insulin delivery, might vary between clinicians in the absence of a uniform, standardised insulin protocol.

## 2 Materials and Methods

### 2.1 Study Design and Setting

This was a bi‐centre cohort study with retrospective data collection of patients acutely admitted to the ICUs at Copenhagen University Hospital—Rigshospitalet and Copenhagen University Hospital—North Zealand. Rigshospitalet is a major tertiary hospital in Copenhagen with a 20‐bed mixed medical and surgical ICU for both adults and children, with approximately 1000 admissions annually, around 100 of which are of paediatric patients. North Zealand Hospital is a large emergency hospital with a 10‐bed mixed medical and surgical ICU, with approximately 600 annual admissions.

The study was conducted and analysed in accordance with a published protocol with minor modifications (Table S1) [18]. The manuscript was reported according to the Strengthening the Reporting of Observational Studies in Epidemiology (STROBE) statement (Table S2).

The study was approved by heads of the two participating ICUs and the legal department at Copenhagen University Hospital—Rigshospitalet. The study was approved as a quality assurance project by the hospital legal authority under Danish law (§42d, stk. 2, nr. 2), permitting waiver of informed consent for retrospective use of routinely collected clinical data.

Patients admitted to the two ICUs were screened for enrolment from 1 December 2023 and backward in time by consecutively backtracking patients admitted to the two ICUs until the target sample size was reached, with the earliest included admission on 31 August 2023. The follow‐up period was 30 days.

### 2.2 Population

Adult patients (≥ 18 years) acutely admitted to either of the ICUs were screened for inclusion. We excluded patients admitted to the ICU for ketoacidosis or hyperosmolar coma; suspected insulin or oral antidiabetic drug intoxication; and patients with severe liver failure (definitions provided in Table S3).

### 2.3 Data Collection and Management

We developed an electronic case report form (eCRF) using the Research Electronic Data Capture (REDCap) [19, 20], hosted by the Capital Region of Denmark. The eCRF was tested by seven medical doctors and researchers, and four nurses at the coordinating site, and subsequently revised before initiation of data collection.

Baseline data were obtained at the time of ICU admission from electronic medical records (Epic/Sundhedsplatformen; Epic Systems Corporation, Verona, WI, USA [21]) and included patient characteristics, presence of sepsis or septic shock, and the Simplified Mortality Score for the Intensive Care Unit (SMS‐ICU; range 0–42, with higher scores indicating higher predicted 90‐day mortality) (definitions in Table S4) [22]. Haemoglobin A1c (HbA1c) values (most recent within 3 months), comorbidities, and chronic medications were automatically extracted from electronic medical records. Daily ICU data were collected from admission through day 30, including blood glucose values, insulin administration, nutritional intake during hypoglycaemic episodes, corticosteroid use, use of life support, and ICU discharge or readmission dates. For patients discharged from the ICU and subsequently readmitted to one of the participating ICUs within 30 days of the index admission, data collection continued. Readmissions to non‐participating ICUs were not captured. A list of variables is provided in Table S5.

### 2.4 Outcomes

The primary outcome was the occurrence of hypoglycaemia (< 4 mmol/L). Secondary outcomes were the occurrence of severe hypoglycaemia (< 2.3 mmol/L), occurrence of hyperglycaemia (> 10 mmol/L), time below target range (< 6 mmol/L), time above target range (> 10 mmol/L), all‐cause mortality at 30 days, the number of days alive without life support (DAWOLS) (i.e., invasive mechanical ventilation, circulatory support, or renal replacement therapy) at 30 days and the number of days alive out of hospital (DAOH) at 30 days. DAWOLS and DAOH at 30 days were reported both as actual (unpenalised) values and as penalised versions, in which death within 30 days was assigned a value of 0 [23]. Process outcomes were days in ICU within 30 days, glucose measurements (number of measurements and method), corticosteroid use in ICU and use of insulin (including route of administration and dosage). Further outcome definitions are described in the Supporting Information.

## 3 Statistical Methods

### 3.1 Sample Size

Assuming a 10% proportion of patients experiencing at least one hypoglycaemic episode during ICU stay [3, 4, 24, 25], a sample of 300 patients would provide a 95% confidence interval of approximately 7%–14% around this estimated occurrence, which we judged sufficient for descriptive precision.

### 3.2 Descriptive Data

Analyses were performed using R version 4.3.2 [26]. Categorical variables were presented as counts and percentages, and continuous variables as medians with interquartile ranges (IQRs), with baseline and outcome data presented for all patients and stratified for diabetes and insulin use. Glycaemic outcomes were assessed amongst patients with at least one recorded glucose measurement. Primary and secondary outcomes were reported descriptively, supplemented with 95% CIs calculated using the Clopper–Pearson method for categorical variables and non‐parametric percentile‐based bootstrapping with 50,000 bootstrap re‐samples for continuous variables.

### 3.3 Regression Analyses

The protocol specified that Cox proportional hazard regression models would be used to assess associations between hyperglycaemia, hypoglycaemia, severe hypoglycaemia, time below target range, and time above target range, and 30‐day mortality, adjusting for potential confounders (SMS‐ICU score, corticosteroid use, diabetes, nutrition, and insulin administration) [18]. However, the numbers of hypoglycaemic and severe hypoglycaemic events were too low to fit the prespecified models. We therefore refrained from the planned regression analyses (Table S1). Mortality at 30 days was therefore reported descriptively stratified for the occurrence of hypoglycaemia, severe hypoglycaemia, and hyperglycaemia.

### 3.4 Linear Interpolation

To estimate time spent outside the target glucose range, we analysed only patients with available glucose and insulin data. Glucose measurements were cleaned by removing identical duplicate records and keeping only the first value when multiple measurements were recorded at the same timestamp. For each patient, glucose values were linearly interpolated at 5‐min intervals between the first and last valid glucose measurement, when at least two valid readings were available. The 5‐min interval was selected to mimic the resolution of continuous glucose monitoring (CGM) data. For each patient, we calculated the proportion of interpolated glucose values below 6 mmol/L and above 10 mmol/L. These proportions were then summarised across the cohort using the median (with CI) and IQR.

### 3.5 Missing Data

Missing data were reported as counts with percentages. Hypoglycaemia was defined based on at least one recorded glucose measurement. Patients with no available glucose values during the ICU stay were assumed not to have experienced hypoglycaemia, on the assumption that no clinical indication for glucose measurement had occurred.

## 4 Results

**FIGURE 1:**
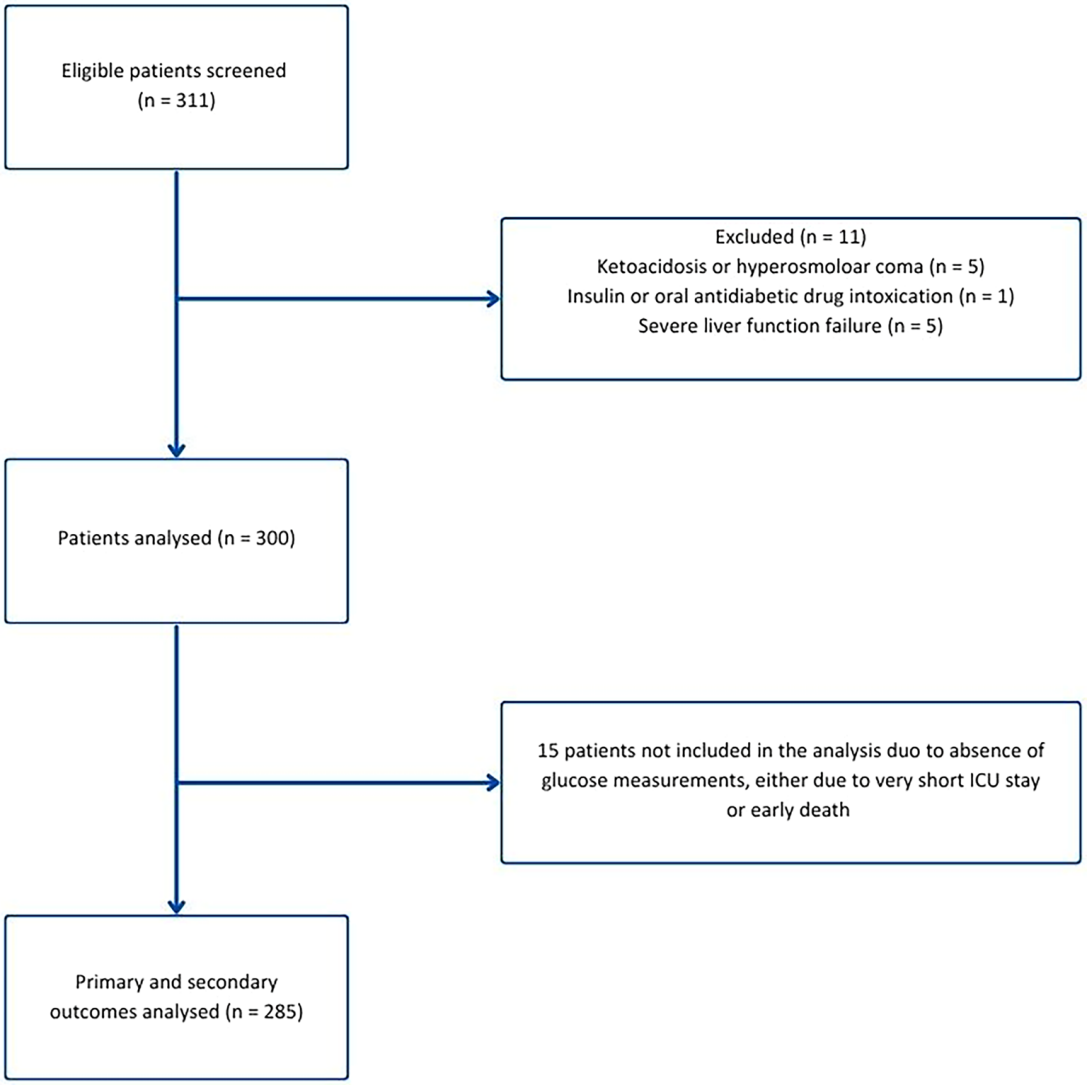
Study flow diagram. Only patients that fulfilled all inclusion criteria were screened for inclusion in the electronic case report form.

A total of 311 acutely admitted adult ICU patients were screened across the two participating Danish ICUs. Eleven patients met an exclusion criterion (Figure 1). Fifteen patients (5% of the cohort) had no glucose measurements, either due to very short ICU stay or early death and were excluded from the outcome analyses.

### 4.1 Baseline Characteristics

Baseline characteristics are summarised in Table 1. The cohort had a median age of 66 years (IQR 55–76), and 41.0% were women. The median SMS‐ICU score at admission was 14 (IQR 10–19), corresponding to an estimated mortality at 90 days risk of approximately 18.4% (IQR 11.6–30.9). Common comorbidities included ischaemic heart disease or heart failure, diabetes and immune deficiency, and a small proportion had received systemic corticosteroids before ICU admission. About half of admissions were medical and half surgical. Most patients were admitted from general wards, with others transferred from emergency departments, operating rooms or other ICUs. At ICU admission, nearly one‐third had sepsis, including a subset with septic shock. Patients with diabetes and those receiving insulin had a higher prevalence of pre‐ICU systemic corticosteroid use, higher median HbA1c, and a slightly greater prevalence of ischaemic heart disease, heart failure, chronic kidney disease, sepsis, and septic shock compared with patients without diabetes and those not receiving insulin in the ICU (Table 1).

**TABLE 1:** Baseline characteristics.

Baseline characteristics	All patients (*N* = 300)	Patients with diabetes (*N* = 63)	Patients without diabetes (*N* = 237)	Patients receiving insulin (*N* = 99)	Patients not receiving insulin (*N* = 201)
Demographics					
Age – median (IQR), years	66 (55–76)	69 (62–76)	65 (49–76)	71 (59–76)	65 (49–76)
Female sex—*n* (%)	123 (41.0)	27 (42.9)	96 (40.5)	39 (39.4)	84 (41.8)
Illness severity—median (IQR)					
SMS‐ICU^a^	14 (10–19)	15 (13–19)	13 (10–18)	16 (13–19)	13 (9–18)
Comorbidities—*n* (%)					
Diabetes	63 (21.0)	63 (100.0)	0 (0.0)	49 (49.5)	14 (7.0)
Ischaemic heart disease or heart failure	58 (19.3)	22 (34.9)	36 (15.2)	26 (26.3)	32 (15.9)
Immune deficiency	41 (13.7)	10 (15.9)	31 (13.1)	18 (18.2)	23 (11.4)
Chronic kidney disease	20 (6.7)	9 (14.3)	11 (4.6)	9 (9.1)	11 (5.5)
Pulmonary disease before ICU	10 (3.3)	2 (3.2)	8 (3.4)	3 (3.0)	7 (3.5)
Pre‐ICU treatments and laboratory values					
Systemic corticosteroids—*n* (%)	23 (7.7)	8 (12.7)	15 (6.3)	12 (12.1)	11 (5.5)
HbA1c—median (IQR), mmol/L^b^	39.0 (35.0–45.2)	43.5 (39.5–51.8)	37.0 (34.0–42.0)	42.0 (35.0–48.0)	38.0 (35.0–42.0)
Admission characteristics—*n* (%)					
Medical admission	154 (51.3)	40 (63.5)	114 (48.1)	59 (59.6)	95 (47.3)
Surgical admission	146 (48.7)	23 (36.5)	123 (51.9)	40 (40.4)	106 (52.7)
Admitted from—*n* (%)^c^					
General ward	117 (44.5)	28 (50.0)	90 (43.3)	44 (50.0)	74 (42.0)
Emergency department	42 (16.0)	10 (17.9)	32 (15.4)	17 (19.3)	25 (14.2)
Operating room	90 (34.2)	16 (28.6)	74 (35.6)	25 (28.4)	65 (36.9)
Other ICU	14 (5.3)	2 (3.6)	12 (5.8)	2 (2.3)	12 (6.8)
Admission diagnoses—*n* (%)					
Sepsis	88 (29.3)	25 (39.6)	63 (26.6)	34 (34.3)	54 (26.9)
Septic shock	46 (15.3)	15 (23.8)	31 (13.1)	20 (20.2)	26 (12.9)
Trauma	24 (8.0)	3 (4.8)	21 (8.9)	4 (4.0)	20 (10.0)

^a^ 90‐day predicted mortality for SMS‐ICU of 14 (IQR: 10–19) is 18.4% (IQR: 11.6–30.9); 15 (IQR: 13–19) is 20.6% (IQR: 16.5–30.9); 13 (IQR: 10–18) is 16.5% (IQR: 11.6–28.0); 16 (IQR: 13–19) is 22.9% (IQR: 16.5–30.9); 13 (IQR: 9–18) is 16.5% (IQR: 10.3–28.0) [22].

^b^ 180 (60%) patients had no measured HbA1c.

^c^ 36 (12%) patients had missing data for admission type variable.

## 5 Outcomes

### 5.1 Primary Outcome

Hypoglycaemia occurred in 21 of 285 patients with at least one recorded glucose measurement, corresponding to 7.4% (95% CI 4.6–11.0). Hypoglycaemia was more frequent in patients with diabetes (11.7%; 95% CI 4.8–22.6) than in those without (6.2%; 95% CI 3.4–10.2) and in those receiving insulin (14.1%; 95% CI 8.0–22.6) than in those not receiving insulin (3.8%; 95% CI 1.5–7.6) (Table 2).

**TABLE 2:** Primary and secondary outcomes.

Outcome	All patients (*N* = 300)	Patients with diabetes (*N* = 63)	Patients without diabetes (*N* = 237)	Patients receiving insulin (*N* = 99)	Patients not receiving insulin (*N* = 201)
Primary outcome—*n* (%; 95% CI)					
Hypoglycaemia^a^	21 (7.4; 4.6–11.0)	7 (11.7; 4.8–22.6)	14 (6.2; 3.4–10.2)	14 (14.1; 8.0–22.6)	7 (3.8; 1.5–7.6)
Secondary glycaemic outcomes					
Severe hypoglycaemia^a^—*n* (%; 95% CI)	3 (1.1; 0.2–3)	1 (1.7; 0.0–8.9)	2 (0.9; 0.1–3.2)	2 (2.0; 0.2–7.1)	1 (0.5; 0.0–3.0)
Hyperglycaemia^a^—*n* (%; 95% CI)	186 (65.3; 59.4–70.8)	57 (95.0; 86.1–99.0)	129 (57.3; 50.6–63.9)	95 (96.0; 90.0–98.9)	91 (48.9; 41.5–56.3)
Time below range^b^, %—median (95% CI; IQR)	—	0.0 (0.0–0.9; 0.0–2.4)	0.8 (0.0–1.9; 0.0–3.4)	0.1 (0.0–0.9; 0.0–2.8)	—
Time above range^b^, %—median (95% CI; IQR)	—	57.0 (34.1–71.1; 23.1–84.8)	37.5 (19.0–55.1; 6.4–68.2)	46.7 (32.8–57.0; 10.0–78.4)	—
Secondary clinical outcomes					
30‐day mortality—*n* (%; 95% CI)	73 (24.3; 19.6–29.6)	13 (20.6; 11.5–32.7)	60 (25.3; 19.9–31.4)	30 (30.3; 21.5–40.4)	43 (21.4; 15.9–27.7)
DAWOLS at 30 days, penalised^c^—median (95% CI; IQR)	27 (26–28; 0–30)	27 (24–28; 12–30)	27 (26–28; 0–30)	24 (21–26; 0–28)	28 (27–29; 3–30)
DAOH at 30 days, penalised^d^, ^e^—median (95% CI; IQR)	9 (2–14; 0–21)	9 (0–14; 0–22)	9 (2–15; 0–21)	0 (0–3; 0–15)	16 (12–18; 0–24)

^a^ 15 patients were not included here due to absence of any recorded glucose measurement.

^b^ Time below range was considered as glucose values < 6 mmol/L, and time above range as glucose values > 10 mmol/L. Outcome assessed amongst patients who received insulin (*n* = 99; 49 with diabetes, 50 without).

^c^ Penalised version assigns 0 DAWOLS at 30 days to patients who died within 30 days [23]. Unpenalised versions are presented in the Table S6.

^d^ Penalised version assigns 0 DAOH at 30 days to patients who died within 30 days [23]. Unpenalised versions are presented in the Table S6.

^e^ Three patients were excluded from the DAOH30 analysis due to unclear admission records.

### 5.2 Secondary Outcomes

Severe hypoglycaemia occurred in 3 of 285 patients (1.1%; 95% CI 0.2–3.0). Hyperglycaemia was common, observed in 65.3% (95% CI 59.4–70.8) of patients. Hyperglycaemia was more frequent in patients with diabetes (95.0%; 95% CI 86.1–99.0) than in those without (57.3%; 95% CI 50.6–63.9) but also in those receiving insulin (96.0%; 95% CI 90.0–98.9) than in those not receiving insulin (48.9%; 95% CI 41.5–56.3). Across the insulin receivers, the median proportion of time below target range was 0.1% (95% CI 0.0–0.9; IQR 0.0–2.8) and the median proportion of time above target range was 46.7% (95% CI 32.8–57.0; IQR 10.0–78.4). Patients with diabetes also had a higher median percentage of time above the target glucose range (Table 2).

Amongst the 285 patients, mortality at 30 days was 24.3% (95% CI 19.6–29.6) (Table 2); 42.8% amongst patients with hypoglycaemia, 66.6% amongst those with severe hypoglycaemia, and 27.9% amongst those with hyperglycaemia (Table 3). The mortality rate was highest amongst patients receiving insulin (30.3%; 95% CI 21.5–40.4). Penalising deaths as zero DAWOLS at 30 days resulted in median of 27 days (95% CI 26–28; IQR 0–30) and DAOH at 30 days median of 9 days (95% CI 2–14; IQR 0–21) (Table 2). Unpenalised values are presented in the Table S6.

**TABLE 3:** 30‐day mortality amongst patients with dysglycaemia.

	Patients with hypoglycaemia (< 4 mmol/L)	Patients with severe hypoglycaemia (< 2.3 mmol/L)	Patients with hyperglycaemia (> 10 mmol/L)
Dysglycaemia^a^, *n*/*N* (%)	21/285 (7.4)	3/285 (1.1)	186/285 (65.3)
Events, *n*/*N* (%)	54/9135 (0.6)	6/9135 (0.1)	2750/9135 (30.1)
Mortality at 30 days, *n*/*N* (%)	9/21 (42.8)	2/3 (66.6)	52/186 (27.9)

^a^ 15 patients were excluded due to absence of any recorded glucose measurement. Percentages are calculated with respect to the total cohort (*N* = 285) for prevalence, and within each subgroup for mortality.

### 5.3 Process Variables

Glucose monitoring was frequent, with a median of 16 (IQR 7–37) measurements per ICU stay per patient and 5 (IQR 3–6) per day per patient. Compared with patients not receiving insulin, patients receiving insulin had a higher number of blood glucose measurements, with a median of 35 (IQR 17–65) per ICU stay and 6 (IQR 5–8) per day. Similarly, patients with dysglycaemia had more measurements than those without, with a median of 24 (IQR 12–52) per ICU stay and 5 (IQR 4–7) per day. Glucose monitoring results stratified by insulin use and dysglycaemia are presented in the Table S7. Nearly all glucose measurements (99.8%) were obtained via point‐of‐care testing, with only 0.2% analysed externally (from the laboratory) (Table 4).

**TABLE 4:** Process outcomes.

Variable	All patients (*N* = 300)
Insulin therapy—*n* (%)	
Patients receiving any insulin	99 (33.0)
Rapid‐acting insulin	99 (33.0)
Long‐acting insulin	4 (1.3)
Insulin route amongst patients receiving insulin^a^, *n* = 99	
Intravenous bolus—*n* (%)	87 (87.9)
Subcutaneous—*n* (%)	38 (38.4)
Insulin infusion—*n* (%)	51 (51.5)
Daily insulin dose per patient, units—median (IQR)^b^	8.5 (3.1–14.9)
Glucose monitoring^c^, *n* = 285	
Glucose measurements per ICU stay—median (IQR)	16 (7–37)
Glucose measurements per day—median (IQR)	5 (3–6)
Method of glucose measurement—*n* (%)	
Point‐of‐care	8186 (99.8)
Laboratory	14 (0.2)
Other ICU interventions—*n* (%)	
Continuous glucose monitoring (CGM)	2 (0.7)
Corticosteroids in ICU	130 (43.3)
Invasive ventilation	174 (58.0)
Vasopressor therapy	87 (29.0)
Continuous renal replacement therapy	22 (7.3)
ICU length of stay	
ICU length of stay, days^d^—median (IQR)	3 (1–6)

^a^ Outcome assessed amongst patients who received insulin (*n* = 99).

^b^ 2 (0.2%) of patients had missing data for insulin dose.

^c^ 15 patients were excluded due to absence of any recorded glucose measurement (*n* = 285).

^d^ Days were rounded up to the nearest whole number.

Amongst the 300 included patients, one‐third (99 patients; 33.0%) received insulin during their ICU stay, all of whom received rapid‐acting insulin and four (1.3%) who also received long‐acting insulin. Intravenous administration was the most common route (87.9% patients). Fifty‐one (51.5%) of the insulin‐treated patients received insulin as a continuous infusion. The median daily insulin dose was 8.5 units (IQR 3.1–14.9) (Table 4).

Other common interventions during ICU stay included corticosteroid therapy (43.3%), invasive ventilation (58.0%), vasopressor use (29.0%), and continuous renal replacement therapy (7.3%). Median ICU length of stay was 3 days (IQR 1–6) (Table 4).

In the subgroup of patients with hypoglycaemia (*n* = 21), 60% received enteral nutrition, 19% received parenteral nutrition and 38.1% received no nutrition. Glucose 50% was administered against hypoglycaemia in 14.3% of these patients, while glucose 20% was not used. Median gastric residual volume on the day of hypoglycaemia in this subgroup was 60 mL (IQR 20–800) (Table 5).

**TABLE 5:** Nutritional and glucose management in patients with hypoglycaemia.

Intervention	*N* = 21
Parenteral nutrition—*n* (%)	4 (19.0)
Enteral nutrition—*n* (%)	12 (60.0)
Glucose 20%—*n* (%)	0 (0.0)
Glucose 50%—*n* (%)	3 (14.3)
Gastric residual volume, mL—median (IQR)^a^	60 (20–800)
No nutrition—*n* (%)	8 (38.1%)

^a^ Gastric residual volume was recorded at the event of hypoglycaemia in 7 of 21 patients (33.3%).

## 6 Discussion

In this bi‐centre cohort of acutely admitted ICU patients, hypoglycaemia occurred in 7.4% of patients, with severe hypoglycaemia being rare (1.1%). Hyperglycaemia was common, affecting nearly two‐thirds of patients in the cohort. Insulin therapy was administered in one‐third of patients, most often via the intravenous route, and glucose monitoring was performed frequently throughout the ICU stay. Patients who received insulin spent almost half of the observed time above the target blood glucose range.

The incidence of hypoglycaemia in our study was higher than that reported in a French retrospective cohort, in which hypoglycaemia (< 3.9 mmol/L) occurred in 2.9% of patients and only one event of severe hypoglycaemia (< 2.3 mmol/L) was observed. Hyperglycaemia (> 10 mmol/L) was less frequent, present in 45.0% of patients, using the same thresholds as in our study [4]. In contrast, another large retrospective cohort of critically ill patients reported hypoglycaemia (< 3.9 mmol/L) in 12.7% and severe hypoglycaemia (< 2.2 mmol/L) in 2.2% of patients [14]. In a post hoc analysis of the *NICE‐SUGAR* randomised trial, hypoglycaemia was more common: in the conventional insulin group, moderate hypoglycaemia (2.3–3.9 mmol/L) occurred in 15.8% of patients and severe hypoglycaemia (≤ 2.2 mmol/L) in 2.3% [27].

Both hypoglycaemia and hyperglycaemia have been associated with worse patient outcomes in critical illness. Hypoglycaemia was associated with increased mortality in the *NICE‐SUGAR* trial in both the tight glycaemic control group and the control group [3, 27]. Hyperglycaemia has also been associated with increased risk of death and prolonged ICU stay in multiple large studies [25, 28, 29, 30, 31]. The patterns observed in our cohort, low occurrence of hypoglycaemia and frequent hyperglycaemia probably reflect the clinical trade‐off of avoiding the first at the expense of the latter but also represent an opportunity for improvement on glucose control in clinical practice.

Insulin therapy was common in our cohort (33%), comparable to the 39% of patients treated with insulin in the conventional‐treatment group of a previous trial [2] and the 45.7% reported in a French cohort [4]. In our study, intravenous administration was the most used, consistent with current guideline recommendations [12, 13]. The American Diabetes Association and the Society of Critical Care Medicine recommend intravenous insulin as the preferred route in critically ill adults, given its reliability under conditions of poor perfusion, vasopressor use or oedema [12, 13].

This cohort study was conducted to inform the planning of a subsequent randomised clinical trial of insulin titration using continuous glucose monitoring versus standard care. The proportion of patients treated with insulin informs recruitment rates and expected enrolment period if the trial is restricted to insulin‐treated patients. In addition, the high frequency of dysglycaemia shows that glucose control is a common clinical problem at these Danish ICUs. Finally, the description of current insulin use informs the development of the insulin protocol that will be used in the trial, while the high frequency of glucose measurements highlights resource use as a relevant outcome.

### 6.1 Strengths and Limitations

Strengths of this study include adherence to a published protocol (with deviations accounted for), transparent reporting in line with STROBE guidelines, and clear documentation of deviations from the protocol when issues arose during data collection.

This study also has limitations. First, it was a retrospective cohort study conducted in two Danish ICUs, which limits the generalisability of the findings. Second, insulin administration data were sometimes difficult to classify as infusion versus bolus, and although consensus was reached with the nursing team, some misclassification may remain. Third, errors in automatic data extraction occurred and while these were corrected where identified, residual inaccuracies cannot be ruled out. Fourth, interpretation of nutrition during hypoglycaemic episodes should be made with caution. In this retrospective design, nutrition was recorded per day rather than at precise time points, meaning that a registration on the same day as hypoglycaemia does not necessarily indicate administration during the episode. Fifth, time below range and time above range values should also be interpreted carefully, as they were extrapolated at 5‐min intervals despite a median of only five glucose measurements per day. Sixth, the observed hypoglycaemia incidence was lower to what we anticipated, and the number of hypoglycaemia (particularly severe hypoglycaemia) events was insufficient to support the multivariable Cox regression models specified in the protocol [18]. Finally, for some variables such as corticosteroid use, gastric residual volume, nutrition, and daily interventions, it was difficult to distinguish between true absence and missing data, as a recorded value confirms use or measurement, but empty fields may reflect either that nothing was done or that it was not documented.

## 7 Conclusions

In this bi‐centre cohort of acutely admitted ICU patients, hypoglycaemia was observed in a minority of patients, while severe hypoglycaemia was rare. Hyperglycaemia was common, affecting more than half of the cohort. Insulin therapy was frequently used, mostly via the intravenous route, and glucose monitoring was performed frequently. Patients who received insulin spent almost half of the observed time above the target glucose range.

## Author Contributions

Milda Grigonyte‐Daraskeviciene, Morten Hylander Møller, Benjamin Skov Kaas‐Hansen, Morten Heiberg Bestle, Christian Lange Gantzel, and Anders Perner conceptualised the study. Milda Grigonyte‐Daraskeviciene and Ruben Julius Eck performed the statistical analyses. Milda Grigonyte‐Daraskeviciene drafted the manuscript with input from the co‐authors and all authors commented on the manuscript. All authors read and approved the final manuscript for publication.

## Funding

The authors have nothing to report.

## Conflicts of Interest

Milda Grigonyte‐Daraskeviciene, Morten Hylander Møller, Benjamin Skov Kaas‐Hansen, Anders Granholm, and Anders Perner are affiliated with the Department of Intensive Care at Rigshospitalet, which has received funding for other projects from the Novo Nordisk Foundation and the Independent Research Fund Denmark. Milda Grigonyte‐Daraskeviciene, Morten Hylander Møller, Benjamin Skov Kaas‐Hansen, Anders Granholm, Morten Heiberg Bestle, Christian Lange Gantzel and Anders Perner are involved in the design of a domain on the Intensive Care Platform Trial (INCEPT, www.incept.dk) investigating continuous glucose monitoring versus usual care. INCEPT is funded by Sygeforsikringen “danmark” and the Novo Nordisk Foundation, and supported by Ehrenreich's Foundation, Dagmar Marshalls Foundation and Savværksejer Jeppe Juhl and Ovita Juhls Mindelegat. Milda Grigonyte‐Daraskeviciene received funding from Ehrenreich's Foundation. Christian Lange Gantzel received a research grant from Research Foundation of North Zealand Hospital.

## Supporting information


**Table S1:** Deviations from data extraction procedures.
**Table S2:** STROBE Statement—checklist of items that should be included in reports of cohort studies.
**Table S3:** Definitions.
**Table S4:** Simplified Mortality Score for the Intensive Care Unit (SMS‐ICU) [5].
**Table S5:** List of variables.
**Table S6:** Secondary outcomes.
**Table S7:** Process outcomes.

## Data Availability

The analysed datasets are not publicly available due to the protection of sensitive patient information. Data may be made available from the corresponding author upon reasonable request and subject to institutional and ethical approvals.
