# Supplementary material for: Glucose Management in the ICU: The Bi‐Centre GEM‐ICU Cohort Study
Source: Acta Anaesthesiol Scand. 2026 Feb 19;70(3):e70207. doi: 10.1111/aas.70207 (PMC12921425; doi:10.1111/aas.70207)
Supplement: Supplementary file 1 — Table S1: Deviations from data extraction procedures. Table S2: STROBE Statement—checklist of items that should be included in reports of cohort studies. Table S3: Definitions. Table S4: Simplified Mortality Score for the Intensive Care Unit (SMS‐ICU) [5]. Table S5: List of variables. Table S6: Secondary outcomes. Table S7: Process outcomes. [file AAS-70-0-s001.pdf]

SUPPORTING INFORMATION

SUPPLEMENT TO:

Glucose management in the ICU: the bi-centre GEM-ICU co-hort study

Milda Grigonyte-Daraskeviciene[1], Ruben Julius Eck[2], Morten Hylander Møller[1,3], Benjamin Skov Kaas-Hansen[1], Morten Heiberg Bestle[3,4], Christian Lange Gantzel[4], Anders Granholm[1,5], Anders Perner[1,3]

[1] Department of Intensive Care, Copenhagen University Hospital – Rigshospitalet, Copenhagen, Denmark

[2] Department of Internal Medicine, University Medical Center Groningen, Groningen, The Netherlands

[3] Department of Clinical Medicine, University of Copenhagen, Copenhagen, Denmark

[4] Department of Anaesthesia and Intensive Care, Copenhagen University Hospital – North Zealand, Hillerød, Denmark

[5] Section of Biostatistics, Department of Public Health, University of Copenhagen, Copenhagen, Denmark

Corresponding author:

Milda Grigonyte-Daraskeviciene, MD

Email: milda.grigonyte-daraskeviciene@regionh.dk

Contents

Deviations from the protocol 3

Table S2. STROBE Statement—checklist of items that should be included in reports of *cohort studies.* 5

Table S3. Definitions 8

Table S4. Simplified Mortality Score for the Intensive Care Unit (SMS-ICU)[3] 13

Table S5. List of variables 15

Outcome definitions 18

Table S6. Secondary outcomes 19

Table S7. Process outcomes 20

References 21

Deviations from the protocol

1.1 Data extraction

Comorbidities, chronic medications, daily and follow-up ICU data were extracted automatically by Centre for IT and Medical Technology (CIMT) service team at the coordinating site. Extracted variables were validated against the electronic health record (EHR). When discrepancies were identified, the study team reviewed the cases and reached consensus on corrections. The underlying cause of these discrepancies could not be fully determined but appeared related to limitations in the initial automated extraction process. The main deviations and corrective actions are summarised in Table S1.

Table S1. Deviations from data extraction procedures

Variable	Error in automated extraction	Correction
**Presence of diabetes (yes/no)**	Incorrect assignment of “yes” or “no” values when compared with EHR records	Variable manually extracted from EHR
**Days with life support**	Start date of life support therapies extracted correctly, but duration in days was not captured	Duration manually extracted from EHR
**Parenteral nutrition**	Start date extracted correctly, but duration in days was not captured	Duration manually extracted from EHR

EHR, electronic health records

1.2 Data reporting

We additionally reported penalised versions of days alive without life support (DAOWLS) and days alive out of hospital (DAOH) at 30 days, although such penalised reporting was not specified in the original protocol.[1]

1.3 Data analysis

The protocol specified that Cox regression would be used to assess associations between hyperglycaemia, hypoglycaemia, severe hypoglycaemia, time below target range, time above target range, and mortality.[2] However, the number of hypoglycaemic and severe hypoglycaemic events was insufficient to reliably fit the planned Cox model. Consequently, these outcomes were reported descriptively with stratification for hypo-/hyperglycaemic events.

Table S2. STROBE Statement—checklist of items that should be included in reports of *cohort studies.*

	Item No.	Recommendation	Page No.
Title and abstract	1	(*a*) Indicate the study's design with a commonly used term in the title or the abstract	1-2
		(*b*) Provide in the abstract an informative and balanced summary of what was done and what was found	2
**Introduction**			
Background/rationale	2	Explain the scientific background and rationale for the investigation being reported	3
Objectives	3	State specific objectives, including any prespecified hypotheses	3
**Methods**			
Study design	4	Present key elements of study design early in the paper	4
Setting	5	Describe the setting, locations, and relevant dates, including periods of recruitment, exposure, follow-up, and data collection	4-5
Participants	6	*(a)* Give the eligibility criteria, and the sources and methods of selection of participants	4, 15
		(b) For matched studies, give matching criteria and number of exposed and unexposed	NA
Variables	7	Clearly define all outcomes, exposures, predictors, potential confounders, and effect modifiers. Give diagnostic criteria, if applicable	5-6
Data sources/ measurement	8*	For each variable of interest, give sources of data and details of methods of assessment (measurement). Describe comparability of assessment methods if there is more than one group	4-6
Bias	9	Describe any efforts to address potential sources of bias	4-7
Study size	10	Explain how the study size was arrived at	5

Quantitative variables	11	Explain how quantitative variables were handled in the analyses. If applicable, describe which groupings were chosen and why	6
Statistical methods	12	(*a*) Describe all statistical methods, including those used to control for confounding	5-7
		(*b*) Describe any methods used to examine subgroups and interactions	16-19
		(*c*) Explain how missing data were addressed	7
		(*d*) If applicable, explain how loss to follow-up was addressed	5
		(*e*) Describe any sensitivity analyses	18
**Results**			
Participants	13*	*(a)* Report numbers of individuals at each stage of study—eg., numbers potentially eligible, examined for eligibility, confirmed eligible, included in the study, completing follow-up, and analysed	15
		(b) Give reasons for non-participation at each stage	8, 15-21
		(c) Consider use of a flow diagram	15
Descriptive data	14*	*(a)* Give characteristics of study participants (eg demographic, clinical, social) and information on exposures and potential confounders	16-17
		(b) Indicate number of participants with missing data for each variable of interest	8, 15-21
		(c) Summarise follow-up time (eg, average and total amount)	4, 18
Outcome data	15*	Report numbers of outcome events or summary measures over time	18
Main results	16	(*a*) Give unadjusted estimates and, if applicable, confounder-adjusted estimates and their precision (e.g., 95% confidence interval). Make clear which confounders were adjusted for and why they were included	16-18
		(*b*) Report category boundaries when continuous variables were categorized	
		(*c*) If relevant, consider translating estimates of relative risk into absolute risk for a meaningful time period	

Other analyses	17	Report other analyses done—eg analyses of subgroups and interactions, and sensitivity analyses	16-19
**Discussion**			
Key results	18	Summarise key results with reference to study objectives	10
Limitations	19	Discuss limitations of the study, taking into account sources of potential bias or imprecision. Discuss both direction and magnitude of any potential bias	11
Interpretation	20	Give a cautious overall interpretation of results considering objectives, limitations, multiplicity of analyses, results from similar studies, and other relevant evidence	10-11
Generalisability	21	Discuss the generalisability (external validity) of the study results	11
**Other information**			
Funding	22	Give the source of funding and the role of the funders for the present study and, if applicable, for the original study on which the present article is based	12

NA – Not applicable
*Give information separately for exposed and unexposed groups.

Note: An Explanation and Elaboration article discusses each checklist item and gives methodological background and published examples of transparent reporting. The STROBE checklist is best used in conjunction with this article (freely available on the Web sites of PLoS Medicine at http://www.plosmedicine.org/, Annals of Internal Medicine at http://www.annals.org/, and Epidemiology at http://www.epidem.com/). Information on the STROBE Initiative is available at www.strobe-statement.org.

Table S3. Definitions

Definitions of eligibility and exclusion criteria
**Ketoacidosis and hyperosmolar coma.** Ketoacidosis defined as plasma glucose >13.9 mmol/l; arterial pH <7,3; serum bicarbonate <18 mmol/l; positive urine ketones and positive serum ketones. Hyperosmolar coma defined as plasma glucose >33.3 mmol/l; arterial pH >7,3; serum bicarbonate >18 mmol/l.
**Insulin or oral antidiabetic drugs intoxication.** Drugs including insulin (human), insulin (beef), insulin (pork), insulin lispro, insulin aspart, insulin glulisine, insulin degludec and insulin aspart, insulin glargine, insulin detemir, insulin degludec, insulin glargine and lixisenatide, insulin degludec and liraglutide, combinations, phenformin, metformin, buformin, glibenclamide, chloropropamide, tolbutamide, glibornuride, tolazamide, carbutamide, glipizide, gliquidone, gliclazide, metahexamide, glisoxepide, glimepiride, acetohexamide, glymidine, combinations, acarbose, miglitol, voglibose, troglitazone, rosiglitazone, pioglitazone, lobeglitazone, sitagliptin, vildagliptin, saxagliptin, alogliptin, linagliptin, gemigliptin, evogliptin, teneligliptin, sitagliptin and simvastatin, gemigliptin and rosuvastatin, exenatide, lixisenatide, albiglutide, dulaglutide, beinaglutiode, dapagliflozin, canagliflozin, empagliflozin, ipragliflozin, sotagliflozin, luseogliflosin, guar gum, repaglinide, nateglinide, pramlintide, benfluorex, mitiglinide, imeglimin, tirzepatide, carfloglitazar, dorzagliatin, tolrestat.
**Severe liver function failure.** Defined as Child-Pugh class C.[3]
**Definitions of outcomes**
**Occurrence of hypoglycaemia.** Number of patients with at least one episode of glucose less than 4.0 mmol/l.
**Occurrence of severe hypoglycaemia.** Number of patients with at least one episode of glucose below or equal to 2.2 mmol/l.
**Occurrence of hyperglycaemia.** Number of patients with at least one episode of glucose above 10 mmol/l.
**Time below target range.** In patients receiving insulin, the percentage of time below the target (6 mmol/l).
**Time above target range.** In patients receiving insulin, the percentage of time above the target (10 mmol/l).
**All-cause mortality at 30 days.** Death within 30 days of ICU admission.
**The number of days alive without life support (i.e., invasive mechanical ventilation, circulatory support, or renal replacement therapy) at 30 days.** Days alive without the use of life-support defined as days alive without the use invasive mechanical ventilation, continuous infusion of vasopressors or inotropic agents and renal replacement therapy (including days between intermittent

haemodialysis).The total number of days alive without the use of life-support will be calculated as the total number of days with the use of life support subtracted from the total follow-up period of 30 days. Patients who died within the 30-day follow-up period were assigned the number of days alive without the use of life-support, calculated accordingly. For the penalised version death within 30 days was assigned a value of 0.

The number of days alive out of hospital at 30 days. Were assessed from the discharge date from the index hospitalization, the number of days readmitted to hospital (if any) and date of death, if relevant, within the 30-day period. The day of discharge did not count, but from the next day at 6 AM. For the penalised version death within 30 days was assigned a value of 0.

Glucose measurements (number of measurements and method). Were assessed daily.

Use of insulin (including route of administration and dosage). Were assessed daily.

Definitions of variables

AT BASELINE

Age. The age of the participant in whole years at the time of admission to ICU. The age was calculated from date of birth.

Sex. Genotypic sex of the patient.

Height. Measured or estimated height in centimetres (cm) at ICU admission.

Weight. Measured or estimated weight in kilograms (kg) at ICU admission.

Date and time of admission to ICU. The time of admission to the ICU the patient was admitted to during the current hospitalization.

Admission type. Surgical admission: all admissions that are originating from the operating or recovery room or if surgical complications lead to ICU admission. Including trauma patients. Medical: any that is not covered in surgical admission will be considered medical irrespective of any surgery done during current ICU admission.

Trauma leading to present hospital admission. Defined as acute accident resulting in injuries to tissue at least one anatomical site or more.

From where was the patient admitted to the ICU?

- **Emergency department or prehospital setting.** Accident/Emergency/Casualty/Acute department in the same or another hospital or direct admission to the ICU by an ambulance service or similar.
- **General ward.** Any location in the same or another hospital not covered in the other 3 categories.
- **Operating or recovery room.** Including surgical room, endoscopy and angiography suite and any recovery facilities observing patients following invasive procedures.
- **Another ICU.** Either within the same or another hospital.

Sepsis or septic shock according to the sepsis-3 criteria within the first 24-hours (no/sepsis/septic shock)? Sepsis: a suspected or confirmed site of infection and an acute change in total SOFA score[4] ≥ 2 points consequent to the infection. Septic shock: a suspected or confirmed site of infection or positive blood culture and ongoing infusion of vasopressor/inotrope agent to maintain a mean arterial blood pressure of 65 mmHg or above and lactate of 2 mmol/L or above in any plasma sample.

Chronic medications. Treatment with prednisolone (≥20mg/day) or other systemic steroid in an equivalent dose, daily for a minimum of 10 days. Other systemic steroid in an equivalent dose includes fludrocortisone >8 mg, betamethasone >2.4 mg, dexamethasone >3 mg, methylprednisolone >16 mg, prednisone >20 mg, triamcinolone >16 mg, hydrocortisone >80 mg, cortisone >100 mg.

Coexisting conditions

- **History of ischemic heart disease or heart failure.** Previous myocardial infarction, invasive intervention for coronary artery disease, stable or unstable angina, NYHA class 3 or 4 or measured LVEF < 40%.
- **Chronic renal failure.** Need for chronic renal support including continuous or intermittent renal replacement therapy or S-creatinine > 300 µmol/L prior to hospital admission.
- **Chronic pulmonary disease.** Treatment at time of hospital admission with any relevant drug indicating chronic pulmonary disease (e.g., COPD, asthma). e.g., salmeterol, formoterol, indacaterol, vilanterol and fluticasone furoate, olodaterol, tiotropium, aclidinium, umeclidinium, glycopyrronium, budesonide and fluticasone.
- **Immune deficiencies. Including:**
    - Solid tumour (active or in remission), solid organ transplant or stem cell transplantation within the last 5 years of index admission.
    - Use of antineoplastic agents including protein kinase inhibitors, monoclonal antibodies and antibody drug conjugates or other antineoplastic agents for more than 30 days.
    - Use of immunosuppressants including selective immunosuppressants, tumour necrosis factor alpha (TNF- α) inhibitors, interleukin inhibitors, calcineurin inhibitors and other immunosuppressants for more than 30 days.
- **Diabetes.** Treatment at time of hospital admission with any relevant drug indicating diabetes e.g., insulins and analogues (A10Ab), blood glucose lowering drugs (A10Bc), other drugs used in diabetes (A10Xd). A10Ab including insulin (human), insulin (beef), insulin (pork), insulin lispro, insulin aspart, insulin glulisine, insulin degludec and insulin aspart, insulin glargine, insulin detemir, insulin degludec, insulin glargine and lixisenatide, insulin degludec and liraglutide, combinations. A10Bc including phenformin, metformin, buformin, glibenclamide, chloropropamide, tolbutamide, glibornuride, tolazamide, carbutamide, glipizide, gliquidone, gliclazide, metahexamide, glisoxepide, glimepiride, acetohexamide, glymidine, combinations, acarbose, miglitol, voglibose, troglitazone, rosiglitazone, pioglitazone, lobeglitazone, sitagliptin, vildagliptin, saxagliptin, alogliptin, linagliptin, gemigliptin, evogliptin, teneligliptin, sitagliptin and simvastatin, gemigliptin and rosuvastatin, exenatide,

lixisenatide, albiglutide, dulaglutide, , beinaglutiode, dapagliflozin, canagliflozin, empagliflozin, ipragliflozin, sotagliflozin, luseogliflosin, guar gum, repaglinide, nateglinide, pramlintide, benfluorex, mitiglinide, imeglimin, tirzepatide, carfloglitazar, dorzagliatin. A10Xd including tolrestat.

Simplified Mortality Score for the Intensive Care Unit (SMS-ICU): details provided in Table S4.

- **Lowest systolic blood pressure (mmHg).** Lowest measured systolic blood pressure in the first 24 hours of admission to ICU.
- **Acute surgical admission.** Acute surgical defined as: if surgical complications lead to ICU admission.
- **Hematologic malignancy or metastatic cancer.** Metastatic cancer defined as proven metastasis by surgery, CT scan or any other method. Haematological malignancy includes any of the following: Leukaemia: Acute lymphoblastic leukaemia (ALL), acute myelogenous leukaemia (AML), chronic myelogenous leukaemia (CML), chronic lymphocytic leukaemia (CLL).; Lymphoma: Hodgkin's disease, and Non-Hodgkin lymphoma (e.g. small lymphocytic lymphoma (SLL), diffuse large B-cell lymphoma, follicular lymphoma and mantle cell lymphoma).; Hairy cell leukaemia (HCL), marginal zone lymphoma, Burkitt's lymphoma, post-transplant lymphoproliferative disorder (PTLD), T-cell prolymphocytic leukaemia (T-PLL), B-cell prolymphocytic leukaemia (B-PLL), Waldenström's macroglobulinemia and other NK- or T-cell lymphomas.; Multiple myeloma/plasma cell myeloma.
- **Use of vasopressors/inotropes.** Any continuous treatment with norepinephrine, epinephrine, phenylephrine, vasopressin analogues, dopamine, dobutamine, milrinone or levosimendan for at least 1 hour during the first 24 hours of admission to ICU.
- **Use of respiratory support.** Including invasive or non-invasive respiratory support and continuous use of continuous positive airway pressure within the 24 hours of admission to ICU (CPAP).
- **Use of renal replacement therapy.** Including any renal replacement therapy whether chronic or acute, continuous renal replacement therapy and intermittent haemodialysis, including the days in between intermittent haemodialysis within 24 hours of admission.

HbA1c value. Reflecting mean blood glucose level over approximately 3 months, measured within 3 months of the index date. If several values within the last 3 months are available, the latest value was considered.

DAY FORM

Use of CGM. Any use of CGM device including GlucoScout; Optiscanner 5000; GlucoDay; CMI system; Sentrino; Guardian REAL-TIME; Guardian 3; Guardian 4; DGMS San; Enlite; GlucoMen Day; FreeStyle Navigator; Freestyle Libre 14 days; Freestyle Libre 2; Freestyle Libre 3; Eversense; Medtrum; Dexcom G4; Dexcom G5; Dexcom G6; Dexcom G7.

Gastric residual volume (ml). The amount aspirated from the stomach following administration of enteral feed.

Rapid-acting insulin. Including insulin (human), insulin (beef), insulin (pork), insulin lispro, insulin aspart, insulin glulisine.

Intermediate-acting insulin. Including insulin (human), insulin (beef), insulin (pork), insulin lispro.

Long-acting insulin. Including insulin (human), insulin (beef), insulin (pork), insulin glargine, insulin detemir, insulin degludec, insulin glargine and lixisenatide, insulin degludec and liraglutide, combinations.

Administration of systemic corticosteroids. Including any dose of hydrocortisone, methylprednisolone, dexamethasone, or prednisolone (IV, IM or oral/per GI tube).

Mechanical ventilation. Invasive mechanical ventilation defined as the use of positive pressure ventilation using a ventilator via a cuffed tube (oral, nasal or tracheostomy). NIV and CPAP were NOT considered invasive mechanical ventilation in the GEM-ICU study.

Use of vasopressors/inotropes. Any continuous (>1 hour) treatment with norepinephrine, epinephrine, phenylephrine, vasopressin analogues, dopamine, dobutamine, milrinone or levosimendan.

Renal-replacement therapy. Any form of renal replacement therapy (e.g., dialysis, hemofiltration or hemodiafiltration) at any rate on this day. Including days between intermittent renal replacement therapy.

FOLLOW-UP

Did the patient die within 30 days after inclusion? Death within 30-day follow-up period.

Has the patient been discharged alive from the hospital within 30 days? Discharged from the hospital and alive within 30-day follow up period. If the patient was discharged alive from the hospital and died at home or at readmission to hospital or ICU it was considered as the patient was discharged alive from the hospital within 30 days.

Has the patient had any additional hospital admissions within 30 days after inclusion? Any readmission to hospital or ICU during 30-day follow-up period.

Table S4. Simplified Mortality Score for the Intensive Care Unit (SMS-ICU)[5]

Simplified Mortality Score for the Intensive Care Unit (SMS – ICU)	
Variable	Points
Age (years)	
≤ 39	0
40 – 59	5
60 – 79	10
≥ 80	13
Lowest systolic blood pressure (mmHg)	
≤ 49	6
50 – 69	5
70 – 89	3
≥ 90	0
Acute surgical admission	
No	3
Yes	0
Haematological malignancy or metastatic cancer	
No	0
Yes	7
Vasopressors / inotropes[a]	
No	0
Yes	4
Respiratory support[b]	
No	0
Yes	5
Renal replacement therapy[c]	
No	0
Yes	4

Total score	0-42[d]

[a] Continuous use of any vasopressor or inotrope.

[b] Use of respiratory support, including invasive or non-invasive respiratory support and continuous use of continuous positive airway pressure (CPAP). Intermittent use of CPAP is not considered respiratory support.

[c] Use of renal replacement therapy includes any renal replacement therapy whether chronic or acute, including continuous renal replacement therapy and intermittent haemodialysis, including the days in between intermittent haemodialysis.

[d] Points assigned for the different variables in the score. It is not possible to obtain a total score of 1, 2 or 40 points. The worst value recorded during the first 24 h in the ICU is used.

Table S5. List of variables

Baseline variables upon admission to ICU
Date of birth
Sex (male/female)
Date and time of admission to ICU
Weight (kg)
Height (cm)
Admission type: surgical/medical
Trauma leading to present hospital admission (yes/no)
From where was the patient admitted to the ICU?<br>o Emergency department or prehospital setting<br>o General ward<br>o Operating or recovery room<br>o Another ICU
Sepsis or septic shock according to the sepsis-3 criteria within the first 24-hours (no/sepsis/septic shock)?
Chronic medications (yes/no)<br>o Treatment with prednisolone (≥20mg/day) or other systemic steroid in an equivalent dose, daily for a minimum of 10 days
Coexisting conditions (yes/no)<br>o History of ischemic heart disease or heart failure<br>o Chronic renal failure<br>o Chronic pulmonary disease<br>o Immune deficiencies<br>o Diabetes
Simplified Mortality Score for the Intensive Care Unit (SMS-ICU):<br>o Lowest systolic blood pressure (mmHg)<br>o Acute surgical admission (no/yes)<br>o Hematologic malignancy or metastatic cancer (no/yes)<br>o Use of vasopressors/inotropes (no/yes)<br>o Use of respiratory support (no/yes)

○ Use of renal replacement therapy (no/yes)
HbA1c value
**Daily during ICU admission (day form)**
Use of CGM on this day (yes/no)
Glucose tests:<br>○ Arterial blood gases (number)<br>○ Capillary blood measures (number)<br>○ Venous blood samples (number)
All blood glucose values (mmol/l)
Nutrition on this day (assessment only at the time of hypoglycaemia):<br>○ Protein or lipid solutions given parenterally (yes/no)<br>○ Enteral nutrition given (yes/no)<br>○ >10% glucose (yes/no)<br>○ Gastric residual volume (ml)
Insulin:<br>Treatment with insulin on this day (yes/no); if yes then:<br>Type of administration:<br>○ IV insulin boluses and S/c insulin (number)<br>○ IV insulin infusion (yes/no)<br>○ SC insulin (yes/no); if yes then:<br>Type of insulin administered:<br>○ Rapid-acting insulin (yes/no)<br>○ Intermediate-acting insulin (yes/no)<br>○ Long-acting insulin (yes/no)<br>Dosage on this day (total international units [IUs])
Administration of systemic corticosteroids on this day (yes/no)
Mechanical ventilation on this day (yes/no)
Use of vasopressors/inotropes on this day (yes/no)
Renal-replacement therapy on this day (yes/no)
**Follow-up**
Did the patient die within 30 days after inclusion? (yes/no)

Has the patient been discharged alive from the hospital within 30 days? (yes/no)
Has the patient had any additional hospital admissions within 30 days after inclusion? (yes/no)<br><br>If yes, then: start and end date of each admission

ICU, intensive care unit

SMS-ICU, simplified mortality score – intensive care unit

HbA1c, haemoglobin A1c

CGM, continuous glucose monitoring

Outcome definitions

DAOH and DAWOLS at 30 days and days in ICU

For the calculation of DAOH at 30 days, DAWOLS at 30 days, and days in ICU, the follow-up period began on the date of ICU admission (day 1). DAOH at 30 days was calculated by subtracting the total number of hospital days from the total number of days alive within 30 days of ICU admission. The maximum possible DAOH at 30 days was 29 days, as day 1 was always spent in hospital. DAOH at 30 days were reported both as unpenalised values and as a penalised version, in which death within 30 days was assigned a value of 0.[1] DAWOLS at 30 days were reported both as unpenalised values and as a penalised version, in which death within 30 days was assigned a value of 0.[1]

Hospital days

Hospital days included the index hospital admission and up to two readmissions within the 30-day follow-up window. Any calendar day on which the patient was hospitalised, including the day of discharge, was counted as a hospital day. Days at home were defined as full days between discharge and any subsequent readmission.

ICU days

ICU days were calculated per patient as the sum of the initial ICU admission and any ICU readmissions within the 30-day follow-up period. Total ICU days were rounded up to the nearest full day and capped at 30 days.

Table S6. Secondary outcomes

Outcome	All patients (N = 300)	Patients with diabetes (N = 63)	Patients without diabetes (N = 237)	Patients receiving insulin (N = 99)	Patients not receiving insulin (N = 201)
**Secondary clinical outcomes**					
**DAWOLS at 30 days, unpenalised- median[a] (95% CI; IQR)**	27 (26 to 28; 8 to 30)	27 (24 to 28; 14 to 30)	27 (26 to 28; 6 to 30)	24 (21 to 26; 5 to 28)	28 (28 to 29; 12 to 30)
**DAOH at 30 days, unpenalised[a,b] - median (95% CI; IQR)**	9 (3 to 14; 0 to 21)	9 (0 to 14; 0 to 22)	9 (3 to 15; 0 to 21)	0 (0 to 3; 0 to 15)	16 (12 to 18; 0 to 24)

[a] Unpenalised version uses the observed value without reassigning a value for death.

[b] Three patients were excluded from the DAOH30 analysis due to unclear admission records.

Table S7. Process outcomes

Outcome	All patients (N = 285)	Patients receiving insulin (N = 99)	Patients not receiving insulin (N = 201)	Patients with dysglycaemia[b] (N = 189)	Patients without dysglycaemia (N = 96)
**Glucose monitoring[a]**					
**Glucose measurements per ICU stay — median (IQR)**	16 (7 to 37)	35 (17 to 65)	11 (5 to 23)	24 (12 to 52)	7 (4 to 14)
**Glucose measurements per day — median (IQR)**	5 (3 to 6)	6 (5 to 8)	4 (3 to 5)	5 (4 to 7)	3 (2 to 5)

[a]15 patients were excluded due to absence of any recorded glucose measurement (n=285).

[b]Dysglycaemia defined as occurrence of any severe hypoglycaemia, hypoglycaemia or hyperglycaemia during ICU stay.

References

1. Granholm A, Kaas-Hansen BS, Lange T, Munch MW, Harhay MO, Zampieri FG, et al. Use of days alive without life support and similar count outcomes in randomised clinical trials – an overview and comparison of methodological choices and analysis methods. BMC Medical Research Methodology. 2023 Jun 14;23(1):139.

2. Grigonyte-Daraskeviciene M, Møller MH, Kaas-Hansen BS, Bestle MH, Nielsen CG, Perner A. Glucose evaluation and management in the ICU (GEM-ICU): Protocol for a bi-centre cohort study. Acta Anaesthesiologica Scandinavica. 2024;68(9):1271–4.

3. Tsoris A, Marlar CA. Use Of The Child Pugh Score In Liver Disease. In: StatPearls [Internet]. Treasure Island (FL): StatPearls Publishing; 2025 [cited 2025 Sep 17]. Available from: http://www.ncbi.nlm.nih.gov/books/NBK542308/

4. Vincent JL, Moreno R, Takala J, Willatts S, De Mendonça A, Bruining H, et al. The SOFA (Sepsis-related Organ Failure Assessment) score to describe organ dysfunction/failure. On behalf of the Working Group on Sepsis-Related Problems of the European Society of Intensive Care Medicine. Intensive Care Med. 1996 Jul;22(7):707–10.

5. Granholm A, Perner A, Krag M, Marker S, Hjortrup PB, Haase N, et al. External validation of the Simplified Mortality Score for the Intensive Care Unit (SMS-ICU). Acta Anaesthesiol Scand. 2019 Oct;63(9):1216–24.
